# Carbonation Perception and Texture Characteristics of Carbonated Beverages Using Focus Group Interviews and Free-Sorting Tasks

**DOI:** 10.3390/foods15112013

**Published:** 2026-06-04

**Authors:** Jihye An, Jeehyun Lee

**Affiliations:** 1 Department of Food Science and Nutrition, Pusan National University, Busan 46241, Republic of Korea; 2 Department of Poultry Science, Auburn University, Auburn, AL 36849, USA; jza0149@auburn.edu

**Keywords:** mouthfeel, FGI, check-all-that-apply, non-expert, sensory attributes

## Abstract

Carbonated beverages are widely consumed worldwide, and carbonation-related texture plays an important role in consumer sensory perception and preference during consumption. This study aims to determine the perception of carbonation and texture characteristics among young Korean adult consumers using a combination of focus group interviews and free-sorting tasks. Participants were asked to describe their experiences with various carbonated beverages, focusing on textural attributes and perceptions. The free-sorting tasks revealed distinct groupings based on perceived carbonation intensity and flavor attributes. At the end of the interview, consumers were presented with a list of texture attribute terms for carbonated beverages and were asked to check all that applied to the essential sensory characteristics of carbonated beverages. The most frequently selected texture attributes were cooling (93.3%, *p* < 0.001), carbonation (86.7%, *p* < 0.001), bite (83.3%, *p* < 0.001), and overall fizziness (76.7%, *p* < 0.01), indicating that these attributes are considered essential by consumers when evaluating carbonated beverages. Interestingly, participants described their carbonation experiences by detailing both their perceived sensations and the precise oral locations at which carbonation, bubble bursting, and tingling attributes were perceived. Overall, this study provides valuable insights into diverse texture perceptions and characteristics of carbonation among young Korean adult consumers.

## 1. Introduction

Soft carbonated beverages are non-alcoholic, sweet, light, flavored, water-based drinks that contain carbon dioxide (CO_2_) and do not include tea, coffee, dairy-based beverages, and alcohol [1]. Carbonated drinks account for the majority of the global soft drink industry and are produced and consumed worldwide [2,3]. According to the Carbonated Soft Drink Global Market Report 2026 [4], the global carbonated beverage market has grown steadily over recent years and is expected to continue growing steadily over the next few years. In line with increasing consumer interest in health, products with zero sugar and low-calorie content are being released. An increase in the consumption of convenient delivered foods has influenced an increase in the consumption of carbonated beverages [5]. Beverages are consumed not only to relieve physiological thirst caused by dehydration, but also for their taste and consumers’ desire to drink them [6]. When people eat or drink, they experience a variety of sensations, including taste, smell, touch, temperature, sight, sound, and sometimes pain or irritation [3].

Carbonation perception is defined as the overall perception of the nasal and oral cavities, including sensations such as stinging, burning, cooling, and irritation [7,8]. The sensations produced by consuming carbonated beverages differ from those of other beverages and are a major hedonic factor contributing to the consumption of carbonated beverages [9]. Trigeminal nerve sensations, such as the tingling sensation caused by CO_2_ in carbonated drinks, may be very important for the overall liking and flavor of foods [10]. Mouthfeel has recently been conceptualized as a multidimensional sensory construct arising from the integration of tactile, trigeminal, and chemesthetic inputs, rather than a single modality perception. This perspective highlights its critical role in shaping consumer acceptance of beverages [11]. Carbonation is a key sensory attribute not only in soft carbonated beverages but also in a wider range of drinks, including alcoholic beverages such as beer [12]. Accordingly, this study aims to investigate carbonation perceptions across diverse carbonated beverages, rather than limiting the scope to soft carbonated drinks.

Research methods in sensory science can be categorized as qualitative and quantitative. In qualitative research, the descriptive analysis method can provide reliable results, but it has the disadvantage of requiring considerable time and money to train panelists and conduct experiments [13]. Therefore, consumer tests are conducted to address these issues. Recently, several studies have shown that descriptive analysis methods targeting trained panels yield similar results to those of consumer research methods [13,14]. However, questions remain regarding whether consumers understand the terminology developed by trained experts [15]. Trained panel members may describe a product differently or consider attributes that are irrelevant to consumers [16]. In particular, consumers often have difficulty employing words related to mouthfeel when describing food [17]. Therefore, it is necessary to examine consumer sensory perceptions and how they express them. Many studies have been conducted concerning the perception of carbonation using the sensations of sight, hearing, pain, and mechanical and chemical senses [18]. Carbonation is a key sensory attribute that influences consumer acceptance and drinking experience [12]. However, few studies have established consumer terminology for the textural characteristics of carbonation. Most existing research has relied on trained panels and expert-derived descriptors, which may not fully reflect how consumers perceive and describe carbonation in real consumption contexts. Understanding consumer-relevant sensory terminology is important for product development and consumer communication. Therefore, it is necessary to investigate the sensory terms used by consumers to describe carbonated beverages and understand how they perceive carbonation.

The objectives of this study were to explore consumers’ perceptions of carbonation and the descriptive terms they use to describe mouthfeel in diverse carbonated beverages, and to determine the criteria by which consumers classify carbonated beverages, as well as the sensory attributes underlying these classifications through a sorting task.

## 2. Materials and Methods

### 2.1. Participants

A total of 30 consumers participated in this study (groups of 5 people per session across 6 sessions; 14 male and 16 female). Courcoux et al. [19] mentioned that when conducting a sorting task with untrained evaluators, having at least 30 participants ensures the stability of the sorting data. The appropriate number of consumers participating in focus groups is five to ten per group [20], with a maximum of <12 for qualitative research [21]. Healthy consumers between 19 and 29 years of age who purchased and consumed carbonated beverages at least once a week were recruited as participants. Applicants were screened and excluded based on the following criteria: individuals who had allergic reactions to carbonated beverages (especially carbonated drinks, carbonated water, ginger ale, and beer) or any food, alcohol sensitivity, caffeine sensitivity (arrhythmia, anxiety, etc.), toothache, difficulty swallowing (dysphagia, etc.), diabetics, vegetarians, or lactating/pregnant women.

### 2.2. Focus Group Interviews (FGIs)

The FGIs were conducted by one moderator and two notetakers who recorded and transcribed the audio recordings. Before starting the interview, participants were informed that the interview content would be recorded. Discussions were conducted in accordance with the moderators’ guidelines (Table 1). First, the session opened with a self-introduction and simple questions [20]: reasons for consuming/purchasing carbonated products, types of carbonated products usually purchased, and sensations or feelings when consuming carbonated beverages. After conducting the free-sorting task [22], additional discussions were held. To derive more diverse sensory terms, consumers were asked to describe the sensory characteristics they experienced when drinking carbonated beverages after consuming samples. They were also asked about the most important sensory characteristics of carbonated beverages, perceived differences in carbonation intensity depending on the type of carbonated beverage packaging, and differences in carbonation perception between original and zero-sugar products.

### 2.3. Free-Sorting Task and Sample Preparation

Fifteen samples with different carbonation intensities and mouth sensations were selected, as shown in Table 2. To capture a wider range of carbonation-related sensory characteristics, two beer samples were included in addition to soft carbonated beverages. Participants were asked to group carbonated beverages based on their similarities (ranging from 2 to 14 groups). After classifying the samples, the consumers were instructed to write down the classification criteria or characteristic terms for each group. All samples were stored in a refrigerator (ETE5107TA-RKR; Electrolux, Seoul, Republic of Korea) until use. Fifteen samples were simultaneously provided to each consumer at the same time in a counterbalanced order, according to the 15 × 15 Williams Latin square design [23]. Samples were provided in the original packaging to minimize loss of carbonation after opening the product. The participants were instructed to open each sample and drink it as usual. Consumers were asked to rinse their mouths with bottled water (330 mL Samdasoo; Kwangdong Pharmaceutical Co., Seoul, Republic of Korea) and eat crackers (100% Farina di Frumento Integrale; Nuova Industria Biscotti Crich S.p.a., Zenson di Piave, Italy) to cleanse their palate between samples. Participants were allowed to re-taste samples as needed and were instructed to take sufficient time to evaluate the samples and complete the sorting task, thereby minimizing potential sensory fatigue. This study was reviewed and approved by the Institutional Review Board at Pusan National University (PNU IRB/2023_161_HR).

### 2.4. Check-All-That-Apply (CATA) with Texture Attributes of Carbonated Beverages

The CATA method was used to identify the essential characteristics of the texture of carbonated beverages. The CATA task was conducted after the sorting task and group discussion, which allowed participants to freely describe the attributes they perceived. Subsequently, a list of attributes was provided, and participants were asked to select the attributes they considered essential when drinking carbonated beverages. All terms used in CATA refer to characteristics related to the texture of carbonic acid (Table 3). Texture attributes related to carbonation were combined with previous studies using sparkling wine [18,24], tonic water [25], carbonated water [8], carbonated beverages [26], regular/diet lemon/lime carbonated beverages [9], kombucha [27], cola and lemon/lime carbonated beverages [17]. At the end of each discussion session, a CATA questionnaire containing texture terms for carbonated beverages was distributed and completed.

### 2.5. Data Analysis

Discussion recorded during the FGIs were transcribed and verified for accuracy. Based on interview transcripts, responses were extracted and analyzed by thematically classifying and clustering similar answers. For the CATA, the frequency of attribute term selection was calculated, and binomial tests were performed to determine whether the selection frequency of each attribute was significantly greater than the level of chance (*p* = 0.5), reflecting attributes perceived as important by participants. The similarity of each consumer’s sorting data was analyzed and visualized in a two-dimensional configuration using multidimensional scaling (MDS) to confirm relationships between samples classified by the sorting task. Agglomerative hierarchical clustering (AHC) was performed to confirm the clusters of samples classified by consumers. MDS and AHC analyses were performed using XLSTAT^®^ software (version 2022.2; Addinsoft Inc., Paris, France).

## 3. Results and Discussion

### 3.1. Reasons Why Consumers Buy/Consume Carbonated Drink (Q1)

Consumers buy or drink carbonated beverages for three main reasons. The first reason was the feeling they could have when consuming it. Consumers responded that they mainly drank carbonated beverages when they wanted to feel cool, refreshed, clean, and thirst-quenched. The second reason was the texture and sensations felt in carbonated drinks. There was an opinion that individuals chose these drinks because of the bite, prickly sensation, irritation, carbonation, and bubbly feeling they have in the mouth. The final reason for this was situational purchases. People responded that they drink carbonated beverages to help with digestion, reduce heavy feelings in the stomach (bloat), and reduce greasy/oily feelings in the mouth.

### 3.2. Sensory Attributes During the Consumption of Carbonated Beverages (Q3-1, Q3-2)

Consumers were asked to respond to questions describing the sensations they experienced while drinking carbonated beverages. Table 4 lists the sensory terms mentioned by the consumers. In this study, sensory terms involving the throat were excluded to focus on mouthfeel. Sensory terms were summarized by categorizing them as mouthfeel during consumption (25), mouthfeel after consumption (10), and effects or feelings in the mouth after swallowing (6).

Consumers mentioned the mouthfeel of carbonated beverages during consumption mainly in terms of carbonation and bubbles. When consumers answered queries concerning carbonation, they also mentioned the bodily location where they felt carbonation (e.g., in the mouth, the tip of the tongue, or throat). It was intriguing that consumers described the sensation along with the location where they felt carbonation and bubbles bursting. In general, the single term “carbonation” is used in the CATA questionnaire for carbonated beverages along with flavor characteristics [9,18,25]. According to a previous study [9], carbonation is defined as the feeling of “small bubbles on the tongue and sides of the mouth while the sample is in the mouth.” This definition of carbonation and what consumers said were very similar, but the location where carbonation was felt more strongly varied depending on the product. This location of sensory perception may also be an important factor regarding whether consumers like the product due to the emotions elicited during the consumption of carbonated beverages.

Various attributes associated with oral pain sensations were identified. Similar to the carbonation attribute, some consumers reported that they experienced a tingling sensation in specific areas, such as both sides of the tongue, the tip of the tongue, and the throat. Even for the same sensory characteristics, the specific location, perception and intensity of pain may differ depending on the sample. This tingling perception, along with the number of bubbles, resulted from the addition of carbonation [28]. Consistent with this, a previous study [26] identified overall fizziness, tingling, drying in the mouth, and irritants as mouthfeel attributes, with carbonation level being the main factor influencing the perception of tingling and irritants. Furthermore, carbonation is known to stimulate the trigeminal system [29], which may explain tingling as an important characteristic of carbonation. In addition, both tingling and numbness have been reported as notable attributes of these drinks “after-sensation” [30].

However, some samples with weak carbonation (particularly beer) are described as having soft and creamy textures. Ivanova et al. [31] used consumer-generated sensory attributes, including mouthfeel, such as watery/thin, astringent/dry, and creamy/smooth/mouth-coating, to evaluate beer. These terms are sensory characteristics that appear as a result of the influence of the carbonated bubble size, and they are one of the factors that can affect whether consumers like carbonated beverages. Recent evidence further suggests that bubble characteristics can influence physiological responses, as smaller and more abundant bubbles have been shown to increase salivary flow and trigeminal activation, thereby enhancing oral perception [29]. In line with this, Lee [32] mentioned that when opening sparkling wine, a more vigorous release of bubbles is associated with richer flavor components, and larger bubbles are perceived to enhance the wine’s deliciousness. Thus, bubble size can be an important factor in consumers’ preference and choice of carbonated products. Depending on the bubble size, carbonated beverages may induce sensory pain or, conversely, create a creamy mouthfeel. These differences may be related to variations in physiological responses induced by bubble structure, including changes in salivation and trigeminal activation [29]. Overall, carbonated beverages are largely evaluated based on bubble-related characteristics, as they play a key role in determining mouthfeel and overall sensory perception [33]. Accordingly, bubble size has been shown to influence consumer preference and perceived quality, with smaller bubbles generally associated with higher acceptability [33]. However, Barker et al. [34] reported that consumers prefer smaller bubbles in carbonated water, whereas Viejo et al. [33] found that consumers tend to dislike samples with bubbles that are too small.

The participants responded to various mouthfeel attributes after drinking carbonated beverages. In particular, they described sensations such as gas expansion, tooth coating, decaying feeling, sugary stickiness (residual), astringency, and shrinking of the mouth. In terms of post-consumption effects, the most frequently mentioned terms were cooling, refreshing, freshness, clean and thirst-quenching. Carbonated mouthfeel can help accelerate the quenching of thirst when compared to still beverages [35]. The observed sensory responses to carbonation may be partly explained by its ability to induce physiological activation, as carbonation has been shown to increase salivation and autonomic responses, thereby enhancing oral perception [29]. However, some contrasting perceptions were also reported. The thirst-inducing attribute was mentioned alongside mouthfeel after consumption, as the coldness induced by trigeminal nerve stimulation may lead to a sensation of oral dryness [36]. Furthermore, many respondents said that carbonated drinks were mainly consumed with oily foods, delivered foods, or fast foods. In this context, attributes, such as refreshing, a feeling of digestion, and cooling, may contribute to the perception that carbonated beverages cleanse greasy or oily residues in the mouth, as reflected in the responses to Q1.

### 3.3. Important Attributes of Carbonated Beverages (Q4)

Through FGIs, the participants responded to questions regarding the characteristics that they considered important in carbonated beverages. Their answers were classified into five categories: carbonation, cooling and freshness, overall fizziness, balance of carbonated beverage flavors, and duration of carbonation.

Most consumers mentioned the sensation of carbonation and its intensity, which is consistent with previous findings that carbonation level plays a critical role in shaping overall sensory perception and consumer response [37]. Although the mention of carbonation intensity varies depending on consumer preferences, the carbonation attribute is commonly mentioned as the most important characteristic. In addition to carbonation, cooling and refreshing were the most frequently mentioned attributes among the various feelings that can be experienced while consuming carbonated beverages.

Consumers mentioned that the overall fizziness of carbonated beverages is an important factor in determining the intensity or amount of carbonation and serves as an indicator of the presence of carbonation when opening carbonated beverages. In this context, auditory cues also play a role. Spence and Wang [38] state that sounds heard before tasting can influence both sensory and hedonic expectations. The fizziness sound of champagne or beer can be used to identify the quality and type of drink, and the carbonation sound produced when pouring into a glass can be used to distinguish between carbonated water and carbonated wine [38,39]. Additionally, the more carbonated bubbles that are heard in sparkling wine, the better the perceived quality control, and the more likely it is that the product has been well fermented [32].

Participants responded that carbonated beverages should have an appropriate balance between carbonation intensity, sweetness, and flavor, supporting previous findings that carbonation level modulates multiple sensory attributes simultaneously, including sweetness perception, aroma, and overall liking [37]. They also answered that when the taste and flavor are mild, weaker carbonation is preferred, whereas stronger carbonation is more suitable for beverages with intense flavor. This perception is consistent with previous studies. In an experiment using a mint-flavored carbonated beverage [28], the addition of CO_2_ increased the aroma intensity, regardless of sucrose concentration. Moreover, the addition of CO_2_ to beverages decreases perceptions of sweetness and increases perceptions of sourness and odor; likewise, carbonated beverages containing sugar are perceived as having reduced freshness [28]. Taken together, these findings suggest that carbonation interacts with multiple sensory attributes, supporting the observed preference for aligning carbonation intensity with strength of flavor [37]. Furthermore, consumer acceptance depends on achieving an optimal level of carbonation, as both excessive and insufficient carbonation can lead to negative perceptions [3]. Therefore, finding a balance between carbonation, sweetness, and flavor is considered an important factor in the development of carbonated beverages, as carbonation intensity acts as a key driver that shapes multiple sensory attributes and overall consumer perception [37].

The duration of carbonation is another important attribute. Once a carbonated beverage is opened and left exposed, carbonation gradually diminishes over time. As carbonated beverages are often not consumed immediately, maintaining carbonation over time becomes an important consideration. This duration may also be related to the degree of carbonation and bubble size. In this regard, previous studies have shown that beverages with small bubbles, such as beer and sparkling wine, generally have longer bubble stability and are preferred by consumers [33,40]. These findings suggest that the perceived duration of carbonation may be closely related to bubble and foam stability, which are key factors influencing sensory perception and consumer acceptability [41].

### 3.4. Categorization of Carbonated Beverages Using Sorting Data (S1)

The participants tested 15 samples and performed a sorting method to determine the characteristics that influenced the classification of carbonated beverages. After sorting, each participant responded to the criteria and reasons for classification. Most participants responded that they were classified based on the flavor and intensity of carbonation.

**Figure 1:**
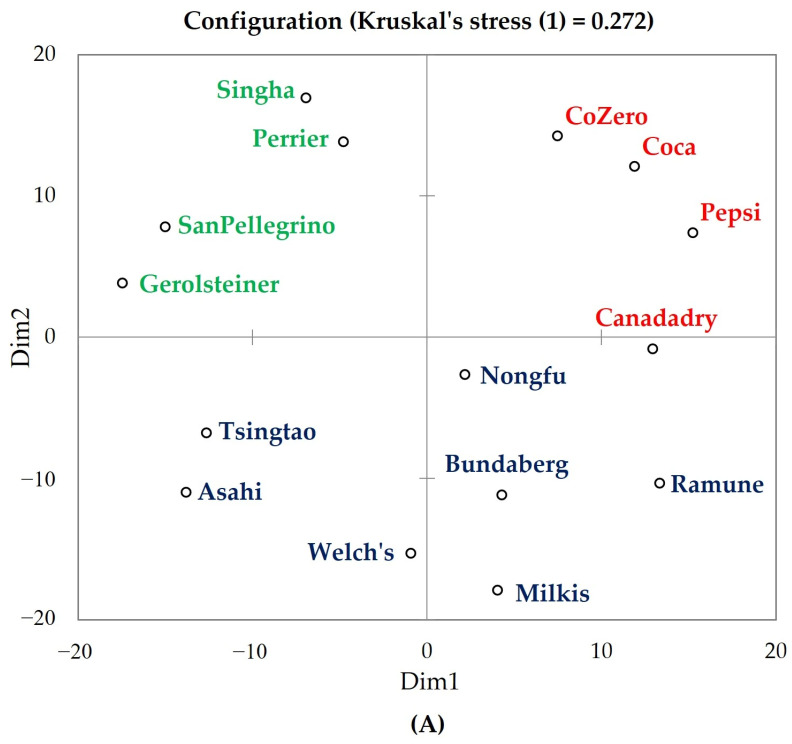

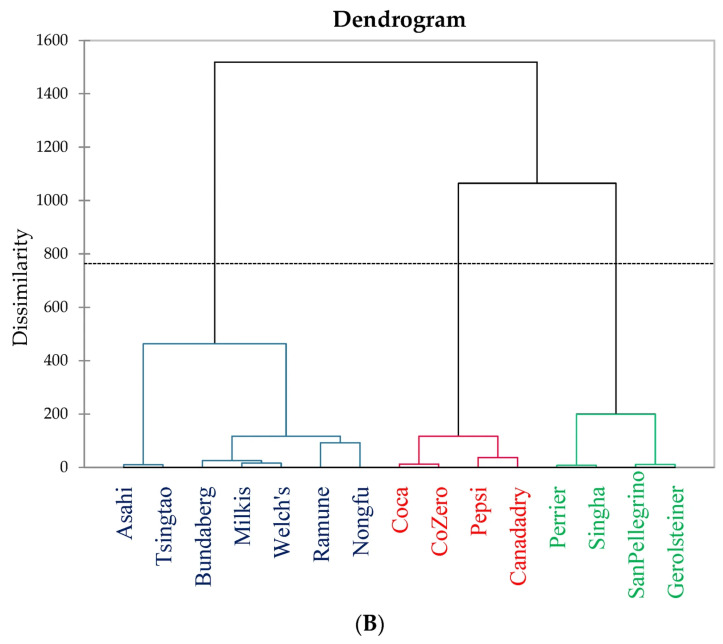
Multidimensional scaling (MDS) map (**A**) and agglomerative hierarchical clustering (AHC) (**B**) of two dimensions using sorting data. Colors represent the three clusters identified by the analysis and are used consistently in both the MDS map and the agglomerative hierarchical clustering dendrogram. Red indicates Group 1 (Cola-type beverages), green indicates Group 2 (carbonated water samples), and blue indicates Group 3 (beer and flavored carbonated beverages).

Based on the sorting data, multidimensional scaling (MDS) analysis was conducted to visualize the relationships among the samples and the attributes associated with their classification (Figure 1). Kruskal’s stress (1) value for the two-dimensional solution was 0.272, indicating a moderate level of fit. Although higher-dimensional solutions showed improved stress values, the two-dimensional configuration was selected to facilitate interpretation and visualization of the relationships among samples and attributes. In Figure 1A, dimension 1 indicates the intensity of the flavor, and dimension 2 indicates the intensity of carbonation. Carbonated water (flavorless) and beer (weak flavor) were located in the negative direction of the x-axis, and relatively flavored products were located in the positive direction. The more the y-axis points toward a positive value, the stronger the carbonation intensity. Singha had the strongest carbonation intensity, whereas Milkis had the weakest. This result shows a similar trend to that in Q2, which asked participants to indicate which products had strong and weak carbonation. In response to Q2, the participants mentioned Coca-Cola (10), carbonated water (10), and Sprite (3) as highly carbonated products. Milkis (13), Pepsi (6), and beer (4) were products with the weakest carbonation. These responses support the configuration of the samples according to carbonation intensity, as shown in Figure 1A. As shown in Figure 1B, the carbonated beverage samples are clearly classified into three groups: Group 1—Cola types (Coca, Cozero, Pepsi, and Canadadry), Group 2—carbonated water (Singha, Perrier, SanPellegrino, and Gerolsteiner), and Group 3—beer and flavored carbonated beverages (Asahi, Tsingtao, Bundaberg, Milkis, Welch’s, Ramune, and Nongfu).

### 3.5. Carbonation Intensity of Original Coke and Coke Zero Sugar (Q7)

As excessive sugar intake leads to various health problems, especially the rising rate of obesity, reducing the intake of added sugar is gaining attention, and the number of low-sugar and sugar alternative beverage products is increasing [42]. The carbonated beverage market has also followed this trend by launching zero-sugar products and attempting to determine whether there is a difference in carbonation intensity for zero-sugar products. Participants were asked to answer questions regarding differences in the intensity of carbonation between Coka and CoZero. Through FGIs, it was confirmed that people had different perceptions of carbonation intensity and liking of Coka and CoZero. Response ratios of the consumers in the current study were as follows: Coka had stronger carbonation (10); CoZero had stronger carbonation (8); the carbonation strengths of the two products were similar (10); and two participants did not respond (2). This result suggests that carbonation perception may vary depending on an individual’s sensory sensitivity. The unique sweetness of each product may influence the perception of carbonation differently in various individuals. The duration of carbonation, the location of feeling, and mouth coating may have influenced how each person evaluated carbonation intensity. For example, if carbonation was strong initially but did not persist for long, or if the carbonation was not strong but lasted for a prolonged time, the evaluation criteria for the intensity of carbonation may differ for each consumer. In a previous study using descriptive analysis [17], Coka showed a significantly higher mouth-coating intensity than CoZero. Most of those who responded that Coka had strong carbonation said that CoZero had strong carbonation initially, but its duration was short owing to its unique artificial sweet taste; therefore, they reported that the original had strong carbonation. The unique artificial sweetness of zero-sugar products is due to the use of various artificial sweeteners, such as aspartame, acesulfame potassium, sucralose, cyclamate, saccharin, etc. [42]. In particular, CoZero contains aspartame and acesulfame potassium (Ace-K) as a sweetener. In a previous study comparing the sweetness of various sweeteners over time using temporal check-all-that-apply [43], aspartame exhibited a slightly wider peak and longer residual sweetness than sucrose. Ace-K showed the highest peak bitterness compared to other artificial sweeteners, which can have unpleasant metallic and chemical tastes [43].

However, blending Ace-K with aspartame improves its sweetness and flavor [44]. As demonstrated in a previous study, where the tingling and stimulation sensations of carbonated beverages were suppressed as glucose increased [26], there may also be a relationship between sweetness, artificial sweeteners, and carbonation. Although this experiment cannot determine how the taste or residual sweetness of these artificial sweeteners affects the sensation or intensity of carbonation, the FGI results showed that consumers’ responses can be subjective, depending on their individual senses and sense interpretation.

### 3.6. Consumer Choices on Essential Sensory Attributes of Carbonated Beverages (C1~3)

After the group discussion, 32 carbonated beverage texture attribute terms were provided to consumers (Table 3), who were asked to check all the attributes that were essential to carbonated beverages. Cooling (28) had the highest response frequency, followed by carbonation (26), bite (25), overall fizziness (23), bubbly (19), after-numbing (18), prickly (17), overall sensation in the mouth (16), and irritant (14). The frequency of attribute selection was analyzed to identify sensory characteristics perceived as important in carbonation. Binomial tests were conducted to determine whether each attribute was selected significantly more frequently than expected by chance (*p* = 0.5). The results showed that several attributes were selected significantly more frequently than level of chance, including cooling (93.3%, *p* < 0.001), carbonation (86.7%, *p* < 0.001), bite (83.3%, *p* < 0.001), and overall fizziness (76.7%, *p* < 0.01). These findings indicate that these attributes represent key terms that consumers consider important when describing carbonated beverages. Participants were asked to indicate which CATA terms they did not understand. They mentioned the feelings of gas expansion (7), pressure (7), and oily/greasy/waxy (5) as the most incomprehensible terms.

Consumers encounter various attribute terms when participating in consumer evaluation using CATA, and the results may be affected by how the terms are interpreted. To examine how consumers interpret sensory terminology, the term “bubble size” was used in this study. Consumers were asked how they interpreted this term and to rate the bubble size of cola and beer on a scale of 1 to 5. The results revealed two distinct interpretation patterns. Nineteen consumers (63.3%) defined bubble size as the size of carbonated particles felt when holding them in the mouth or the size of the bubbles felt in their mouth and accordingly reported that cola had a larger bubble size than beer. This interpretation is consistent with Harper and McDaniel [8], who defined bubble size as “the perception of the size of the bubbles in the mouth.” In contrast, 11 consumers (36.7%) interpreted bubble size as the volume of visible foam and reported that beer had a larger bubble size than cola. This perspective is also supported by previous research showing that the amount of visible bubble formation can be a useful indicator of the perceived presence of bubbles in the mouth [12]. These findings suggest that consumers may rely on both visual and oral cues when interpreting carbonation-related attributes, which may contribute to variability in how sensory terms are understood across individuals. Therefore, the sensory terms used in consumer evaluations may not be interpreted consistently, highlighting the need for careful consideration when applying CATA terms.

To address this issue, the use of appropriate and precise terminology is necessary to minimize this error. For example, Langstaff et al. [45] conducted a sensory evaluation of beer mouthfeel by distinguishing between “bubble size” and “foam volume” as separate attributes. While sensory terms and their definitions are typically standardized through the agreement of expert panels, additional efforts are required to minimize discrepancies in interpretation when such terms are presented directly to consumers. Reducing these gaps in terminology interpretation may ultimately improve the reliability of sensory evaluation results.

## 4. Conclusions

This study confirmed consumers’ perceptions of carbonated beverages and the sensory characteristics they described through FGIs and identified the criteria for classifying carbonated beverages and the criteria influencing the classification criteria through sorting tasks. Because this experiment involved a sorting task using various types of carbonated beverages, the beverages tended to be classified by broad characteristics (carbonation intensity and flavor). If sorting tasks were conducted with more specific types of carbonated beverages, the classification criteria would be slightly more refined.

In addition, when using the CATA evaluation form with sensory terms developed by experts, it was confirmed that there may be differences in consumer interpretations of sensory attribute terms, which may influence the analysis and outcomes of sensory evaluation data. To minimize such variability, experimental conditions, which may affect consumers during sensory evaluation (e.g., locations, temperature, pressure, humidity, and lighting), should be controlled. Furthermore, it is important that the terms used in sensory questionnaires are presented under consistent definition conditions for consumers. These findings provide useful insights for the development of carbonated beverages and for improving communication through consumer-relevant sensory terminology.

This study has several limitations. First, the participant sample was limited to young Korean adults who regularly consume carbonated beverages, which may restrict the generalization of the findings. This is because carbonation perception may vary depending on age, consumption frequency, and cultural backgrounds. Therefore, caution is needed when extending these results to broader populations. Second, this study focused on consumer perception without incorporating instrumental measurements of carbonation properties. Since sensory attributes such as carbonation intensity, bubble size, and duration of carbonation are influenced by physical factors, the relationship between perceived attributes and underlying physical properties could not be directly established.

Future research should address these limitations by including more diverse consumer groups to improve generalizability. In addition, integrating instrumental analysis with consumer-based sensory evaluation would provide a more comprehensive understanding of how physical properties of carbonation influence consumers’ perception.

## Figures and Tables

**Table 1:** Summary of moderator’s guide and participant questions ^(1)^.

Stage	Questions
Warm-up	Self-introduction
Introduction	[Q1] Why do you purchase/consume carbonated drinks? [Q2] What carbonated beverages do you usually drink? And among the products you usually drink, which products are lightly/strongly carbonated? [Q3-1] What characteristics do you feel when you think of or drink carbonated beverages?
Sorting task	Taste the 15 samples provided and sort them into groups you think are similar. [S1] How did you classify the samples and what were your criteria for grouping them?
Discussion	[Q3-2] What sensory characteristics do you feel when you drink carbonated beverages? [Q4] What sensory characteristics do you think are important in carbonated beverages? [Q5] Which sample had the right carbonation intensity for you? [Q6] Do you feel a difference in carbonation intensity between CAN/PET/GLASS packages? Which sample had the highest perceived carbonation intensity? And which sample was most liked? [Q7] Is there a difference in carbonation intensity between original Coke and Coke Zero Sugar? If so, what sensory characteristics were different?
CATA	Among the texture attributes of the carbonated beverages provided, please check all essential characteristic terms that you feel when drinking the carbonated beverage. [C1] What attribute terms did you choose and why? [C2] Were there any terms you didn’t understand? [C3] When you see the term “Bubble size,” what does it mean to you?
Ending	Ask for other comments/suggestions that were not answered during the interview

^(1)^ Abbreviations: CATA = check-all-that-apply; PET = polyethylene terephthalate; CAN = aluminum can.

**Table 2:** Sample information of carbonated beverages (alphabetical order).

Sample	Product Name	Company	City	Country	Amount (mL)	Packaging
Asahi	Asahi Superdry Beer	Asahi Breweries, Ltd.	Tokyo	Japan	500	CAN
Bundaberg	Bundaberg Pink Grapefruit Sparkling Drink	Bundaberg Brewed Drinks PTY Ltd.	Bundaberg	Australia	375	Glass
Canadadry	Canada Dry Ginger Ale	Coca-Cola Beverage Company.	Seoul	Republic of Korea	250	CAN
Coca	Coca-Cola	Coca-Cola Beverage Company.	Seoul	Republic of Korea	300	PET
CoZero	Coca-Cola Zero Sugar	Coca-Cola Beverage Company.	Seoul	Republic of Korea	300	PET
Gerolsteiner	Gerolsteiner Sparkling Natural Mineral Water	Gerolsteiner Brunnen GmbH & Co.	Gerolstein	Germany	500	PET
Milkis	Milkis	Lotte Chilsung Beverage Co., Ltd.	Seoul	Republic of Korea	500	PET
Nongfu	Nongfu Spring Sparkling Tea Drink (Citrus pu’re)	Nongfu Spring	Hangzhou	China	470	PET
Pepsi	Pepsi Cola	Lotte Chilsung Beverage Co., Ltd.	Seoul	Republic of Korea	600	PET
Perrier	Perrier Original	Nestle waters supply SUD	Vergèze	France	330	Glass
Ramune	Sangaria Ramune	Japan Sangaria Beverage Co., Ltd.	Osaka	Japan	190	CAN
SanPellegrino	San Pellegrino Sparkling Water	Sanpellegrino S.P.A,	San Pellegrino Terme	Italy	250	Glass
Singha	Singha Soda Water	Wangnoi Beverage Co., Ltd.	Wang Noi	Thailand	325	Glass
Tsingtao	Tsingtao beer	Tsingtao Brewery Co., Ltd.	Qingdao	China	500	CAN
Welch’s	Welch’s Sparkling Grape Soda	Nongshim Co., Ltd.	Seoul	Republic of Korea	300	PET

**Table 3:** Texture attribute terms of carbonated beverages ^(1)^.

**Before consuming (2)**		
Overall fizziness ^3^	Foamy ^5,8^	
**During consumption (28)**		
Overall sensation in the mouth ^5^	Foam stability ^6^	Bubble size ^2^
Bubble sound ^2^	Bubbly ^2^	Bite ^2,4,6,7,8^
Burn ^2,4,5,6,7,8^	Bubble pain ^8^	Irritant ^3^
Numbing ^2,4,6,7,8^	Prickly ^8^	Tingly ^3,8^
Nasal pungency ^5^	Gas expansion feeling ^2^	Pressure ^8^
Carbonation ^1,4,5,6,7,8^	Chalky ^2^	Light ^1^
Cooling ^2^	Mint ^1^	Slipperiness/sliminess ^6^
Oily/greasy/waxy ^5^	Body ^4^	Creamy ^5^
Mouth coating ^4,5,6^	Tongue heaviness ^6,7^	Tooth etching ^6^
Drying in mouth ^3^		
**After swallowing (2)**		
After-numbing ^6,8^	Astringency ^2,4,5,6,7^	

^(1)^ Superscripts indicate the original sources from which each sensory term was adopted: ^1^ Alderson et al. (2021) [27] and ^2^ Harper and McDaniel (1993) [8]; ^3^ Hewson et al. (2009) [26]; ^4^ Kappes et al. (2006) [17]; ^5^ Le Barbé (2014) [24]; ^6^ Leksrisompong et al. (2012) [9]; ^7^ Marjanska & Szpakowska (2020) [25]; ^8^ McMahon et al. (2017) [18].

**Table 4:** Terms related to texture attributes of carbonated beverages mentioned by consumers.

**Mouthfeel during consuming carbonated beverages (25)**		
Carbonation (in mouth) * ^(1)^	Carbonation (on the tip of the tongue)	Small and dense carbonation
Chalky/carbonation particles *	Bubble	Bubbly *
Bubble bursting	Bubble bursting (on the tip of the tongue)	Bubble pressure *
Air bubble	Foamy	Creamy *
Soft	Light *	Bite *
Numbing *	Pungency	Nasal pungency *
Tingling (in mouth) *	Tingling (on both sides of the tongue)	Tingling (on the tip of the tongue)
Prickling *	Bubble pain *	Burn*
Irritant *		
**Mouthfeel after consuming carbonated beverages (10)**		
Mouth coating *	Tooth coating	Tooth etching (decaying) *
Gas expansion feeling *	Sticky/thickness (residual)	Oil-coating
Thirst-inducing	Astringency *	Shrinking (inside cheeks)
Drying in mouth *		
**Effect or feeling in the mouth after swallowing (6)**		
Cooling *	Cold	Refreshing
Freshness	Clean	Thirst-quenching

^(1)^ The “*” mark indicates overlap with the lexicon attributes of the CATA questionnaire (Table 3).

## Data Availability

The original contributions of this study are included in the article. Further inquiries can be directed to the corresponding author.

## Abbreviations

The following abbreviations are used in this manuscript:

CATA	Check-all-that-apply
FGI	Focus group interview
Ace-K	Acesulfame potassium
